# Tissue-resident M2 macrophages directly contact primary sensory neurons in the sensory ganglia after nerve injury

**DOI:** 10.1186/s12974-021-02283-z

**Published:** 2021-10-13

**Authors:** Haruki Iwai, Koji Ataka, Hajime Suzuki, Ashis Dhar, Eriko Kuramoto, Atsushi Yamanaka, Tetsuya Goto

**Affiliations:** 1 Department of Oral Anatomy and Cell Biology, Graduate School of Medical and Dental Sciences, Kagoshima University, 8-35-1 Sakuragaoka, Kagoshima, Kagoshima 890-8544 Japan; 2 Department of Psychosomatic Internal Medicine, Graduate School of Medical and Dental Sciences, Kagoshima University, 8-35-1 Sakuragaoka, Kagoshima, Kagoshima 890-8544 Japan; 3 Laboratory of Medical Biochemistry, Kobe Pharmaceutical University, 4-19-1 Motoyamakita-machi, Higashinada-ku, Kobe, 658-8558 Japan; 4 Department of Oral and Maxillofacial Surgery, Graduate School of Medical and Dental Sciences, Kagoshima University, 8-35-1 Sakuragaoka, Kagoshima, Kagoshima 890-8544 Japan

**Keywords:** Neuroinflammation, Nerve injury, Bone-marrow-derived macrophage, Tissue-resident macrophage, M1, M2, Cell contact, Sensory ganglion, Primary sensory neuron, Satellite glial cell

## Abstract

**Background:**

Macrophages in the peripheral nervous system are key players in the repair of nerve tissue and the development of neuropathic pain due to peripheral nerve injury. However, there is a lack of information on the origin and morphological features of macrophages in sensory ganglia after peripheral nerve injury, unlike those in the brain and spinal cord. We analyzed the origin and morphological features of sensory ganglionic macrophages after nerve ligation or transection using wild-type mice and mice with bone-marrow cell transplants.

**Methods:**

After protecting the head of C57BL/6J mice with lead caps, they were irradiated and transplanted with bone-marrow-derived cells from GFP transgenic mice. The infraorbital nerve of a branch of the trigeminal nerve of wild-type mice was ligated or the infraorbital nerve of GFP-positive bone-marrow-cell-transplanted mice was transected. After immunostaining the trigeminal ganglion, the structures of the ganglionic macrophages, neurons, and satellite glial cells were analyzed using two-dimensional or three-dimensional images.

**Results:**

The number of damaged neurons in the trigeminal ganglion increased from day 1 after infraorbital nerve ligation. Ganglionic macrophages proliferated from days 3 to 5. Furthermore, the numbers of macrophages increased from days 3 to 15. Bone-marrow-derived macrophages increased on day 7 after the infraorbital nerve was transected in the trigeminal ganglion of GFP-positive bone-marrow-cell-transplanted mice but most of the ganglionic macrophages were composed of tissue-resident cells. On day 7 after infraorbital nerve ligation, ganglionic macrophages increased in volume, extended their processes between the neurons and satellite glial cells, and contacted these neurons. Most of the ganglionic macrophages showed an M2 phenotype when contact was observed, and little neuronal cell death occurred.

**Conclusion:**

Most of the macrophages that appear after a nerve injury are tissue-resident, and these make direct contact with damaged neurons that act in a tissue-protective manner in the M2 phenotype. These results imply that tissue-resident macrophages signal to neurons directly through physical contact.

**Supplementary Information:**

The online version contains supplementary material available at 10.1186/s12974-021-02283-z.

## Background

Macrophages are distributed in the peripheral nervous system and are key players in the repair of nerve tissue and the development of neuropathic pain caused by peripheral nerve injury. They are activated after nerve injury, which leads to nerve fiber repair and hyperalgesia [1, 2]. By contrast, suppression of macrophages, such as pharmacological manipulation, suppresses hyperalgesia [3] and delays the process of nerve fiber repair, such as Wallerian degeneration after nerve injury [4]. Macrophages occupy nerve fibers and sensory ganglia in the peripheral nervous system [1]. Those located at the site of the nerve fiber injury as well as those in sensory ganglia away from the site are activated on nerve fiber injury [1]. For instance, ganglionic macrophages positive for ionized calcium-binding adaptor molecule 1 (Iba1) proliferate [5] and increase in number after nerve injury [6, 7].

Macrophages are broadly divided into tissue-resident macrophages and bone-marrow-derived (BMD) macrophages [8]. Studies on green-fluorescent-protein-positive bone-marrow-cell-transplanted (GFP-BMT) mice have shown that after nerve injury the spinal dorsal horn in the central nervous system is infiltrated not only by microglia, a tissue-resident macrophage, but also by BMD macrophages [9, 10]. Moreover, a study of physiological turnover reported that approximately 80% of BMD macrophages in sensory ganglia are replaced within 3 months [11]. However, radiation results in infiltration of BMD macrophages into the spinal cord dorsal horn [12], which may also occur in sensory ganglia. A method to protect the mouse brain from radiation damage has been devised, namely, placing a lead cap on the mouse’s head [13, 14]. We thought it would be useful to observe the trigeminal ganglion located in the lower part of the brain using GFP-BMT mice with the head protected by a lead cap to distinguish tissue-resident macrophages in the ganglion from BMD macrophages after nerve injury.

The morphology of macrophages differs depending on tissue and can change depending on activation state. Microglia in the central nervous system have a ramified process in the normal state and are in a rod or amoeboid shape in the pathological state [15]. Macrophages in the sensory ganglia are amoeboid, similar to microglia in the central nervous system, in a mouse model of hemiplegic migraine [16]. On the other hand, there are different reports that these macrophages are amoeboid in normal conditions and become stellate after nerve injury [17]. Furthermore, ganglionic macrophages become ring-like and surround damaged neurons in nerve-injury models [18, 19]. In addition, ganglionic macrophages contact neurons after nerve injury [6]. As neurons in healthy sensory ganglia are covered with glutamine synthetase-positive satellite glial cells [20], the morphological relationships among macrophages, neurons, and satellite glial cells need to be clarified in three dimensions if macrophages contact neurons after nerve injury.

Macrophages can be classified into either the M1 pro-inflammatory phenotype, which secretes cytokines, or the M2 anti-inflammatory phenotype, which protects tissues [21]. After nerve injury, ganglionic macrophages increase levels of the M1 marker cluster of differentiation (CD) 86, the M2 marker CD206 [22], or CD206 alone [7, 23, 24]. However, it is unclear whether macrophages should be classified as the M1 or M2 phenotype and phagocytose neurons when they come in contact with neurons.

In this study, we clarified the origin and three-dimensional morphological changes in macrophages after chronologically organizing their activation patterns in sensory ganglia after nerve injury. We examined when macrophages reach their peak of division, whether tissue-resident or BMD macrophages dominate, what individual forms the macrophages take and how they contact damaged neurons, whether macrophages are classified as the M1 or M2 phenotype after contact, and whether neural cell death occurs. We found that M2 macrophages, which are found mainly in sensory ganglia, proliferate, increase in volume, and enter areas between primary sensory neurons and satellite glial cells, which make direct contact with neurons after nerve injury.

## Methods

### Animals

This study was approved by the Animal Experiment Committee of Kagoshima University and was conducted according to the National Institutes of Health (NIH) Guide for the Care and Use of Laboratory Animals. All efforts were made to minimize the number of animals included. We used 79 male C57BL/6J mice weighing 20–30 g (Japan SLC, Shizuoka, Japan) and C57BL/6-Tg (UBC-GFP) 30Scha/J (green-fluorescent-protein-transgenic; GFP-Tg) mice (Jackson Laboratory, Bar Harbor, ME, USA). Surgery was performed under anesthesia induced by an intraperitoneal injection of 0.3 mg/kg medetomidine (Meiji Seika Pharma, Tokyo, Japan), 4.0 mg/kg midazolam (Novartis, Basel, Switzerland), and 5.0 mg/kg butorphanol (Meiji Seika Pharma).

### Bone-marrow transplantation

Bone-marrow transplantation was performed according to previous studies [13, 14]. Bone-marrow cells were isolated from the femurs and tibias of GFP-Tg mice (*n* = 3). Recipient 7–9-week-old C57BL/6J mice (*n* = 7) with the head covered with a lead cap received whole-body irradiation of 10 Gy, and bone-marrow cells (4–6 × 10^6^) were injected into their tail veins. GFP-BMT mice were maintained in cages, covered with filter caps, and given sterile water including 0.001 N HCl (pH 2.0) and sterile chow for 2 weeks to prevent infection. Eight-to-ten weeks after transplantation, the ratio of GFP-positive cells in monocytes was examined in each mouse by flow-assisted cell sorting (Accuri C6; BD Biosciences, Franklin Lakes, NJ, USA). GFP-BMT mice with a chimeric ratio > 70% were used.

### Animal surgery

Animal surgery was performed according to a previous study [25]. In 57 mice (8–12-week-old wild-type mice, *n* = 50; 7-month-old GFP-BMT mice, *n* = 7), the left side of the infraorbital nerve was the experimental side and the right side was the sham-operated side. Nineteen mice were used as naïve controls. As a previous study using GFP-BMT mice reported that 80% of the physiological turnover of macrophages in sensory ganglia occurred within 3 months [11], we used 7-month-old mice, which was 5 months after the transplantation. The buccal mucosa of the upper jaw on the left experimental side was small incised and the infraorbital nerve came out from the foramen was exposed. The nerve was tightly ligated with 7-0 silk suture in wild-type mice. As a previous study using GFP-BMT mice prepared a nerve crush lesion model [11], the infraorbital nerve was transected to completely damage the entire nerve rather than part of it in the GFP-BMT mice. After nerve injury, the incision was closed with tissue adhesive. On the right sham-operated side, the infraorbital nerve received the same procedure on the left side without ligature or transection. After a survival period of 1 day to 4 weeks, the mice were deeply anesthetized, transcardially perfused with phosphate-buffered saline (PBS; pH 7.3), and fixed in 4% formaldehyde in phosphate buffer (pH 7.3) for immunohistochemistry or in 4% paraformaldehyde and 0.05% glutaraldehyde in phosphate buffer (pH 7.3) for electron microscopy. Forty-six mice received an intraperitoneal injection of 100 mg/kg bromodeoxyuridine (BrdU; B5002, Merck, Darmstadt, Germany) in PBS to detect cell proliferation at 24 h before perfusion.

### Immunohistochemistry

The trigeminal ganglion was removed, postfixed in the same fixative solution, kept overnight at 4 °C, and immersed in 30% sucrose in PBS for 2–3 days at 4 °C. Serial horizontal sections of two thicknesses of the trigeminal ganglion were cut on a cryostat (CryoStar NX70; Thermo Fisher Scientific, Waltham, MA, USA); 30-μm sections were used for normal histological observation, and 50-μm sections were used for three-dimensional reconstructions. Immunostaining was divided into two groups, one for bright-field observations and the other for fluorescence observations, and each was reacted at room temperature. The sections for BrdU immunostaining were submerged in 2 N HCl for 60 min before the immunoreaction.

The sections were incubated with 1% bovine serum albumin and 0.2% Triton X-100 in PBS for 1 h before the fluorescence observations; primary antibodies were incubated overnight in the same incubation buffer, and secondary antibodies were incubated in the same incubation buffer for 3 h. Subsequently, the sections received nuclear staining of 1:100 4ʹ,6-diamidino-2-phenylindole (DAPI; 340-07971, Dojindo, Kumamoto, Japan) or Nissl staining with 1:1000 NeuroTrace 500/525 (N-21480, Thermo Fisher Scientific) in PBS. The antibodies used are shown in Table 1.

**Table 1:** Antibodies used in the present study

	Catalog no	Host	Clonality	Concentration	Manufacturer	RRID
Primary antibody						
Anti-activating transcription factor 3 (ATF3) (a marker of damaged neuron)	HPA001562	Rabbit	Polyclonal	1:1000	Atlas Antibodies, Stockholm, Sweden	AB_1078233
Anti-bromodeoxyuridine (BrdU) (a marker of cell-proliferation)	ab6326	Rat	Monoclonal	1:1000	Abcam, Cambridge, UK	AB_305426
Anti-ionized calcium binding adaptor molecule 1 (Iba1) (a marker of macrophage/microglia)	ab5076	Goat	Polyclonal	1:5000	Abcam, Cambridge, UK	AB_2224402
Anti-product gene protein 9.5 (PGP9.5) (a marker of neuron)	GP14104	Guinea pig	Polyclonal antiserum	1:5000	Neuromics, Edina, MN	AB_2210625
Anti-caspase-3 (a marker of cell death)	NB600-1235	Rabbit	Polyclonal	1:500	Novus Biologicals, Littleton, CO	AB_2069897
Anti-glutamine synthetase (a marker of satellite glial cell)	G2781	Rabbit	Polyclonal	1:10,000	Merck, Darmstadt, Germany	AB_259853
Anti-mouse cluster of differentiation 206 (CD206) (a marker of M2 phenotype)	AF2535	Goat	Polyclonal	1:1000	R&D Systems, Minneapolis, MN	AB_2063012
Anti-mouse cluster of differentiation 86 (CD86) (a marker of M1 phenotype)	553,689	Rat	Monoclonal	1:1000	R&D Systems, Minneapolis, MN	AB_394991
Secondary antibody						
Anti-rat IgG-conjugated to Alexa Fluor 488	ab150153	Donkey	Polyclonal	1:800	Abcam, Cambridge, UK	AB_2737355
Anti-rabbit IgG-conjugated to Alexa Fluor 488	ab150065	Donkey	Polyclonal	1:800	Abcam, Cambridge, UK	AB_2860569
Anti-rabbit IgG-conjugated to Alexa Fluor 555	ab150066	Donkey	Polyclonal	1:800	Abcam, Cambridge, UK	
Anti-rabbit IgG-conjugated to Alexa Fluor 647	ab150067	Donkey	Polyclonal	1:800	Abcam, Cambridge, UK	
Anti-goat IgG-conjugated to Alexa Fluor 555	A-21432	Donkey	Polyclonal	1:800	Thermo Fisher Scientific, Waltham, MA	AB_2535853
Anti-goat IgG-conjugated to Alexa Fluor 647	A-21447	Donkey	Polyclonal	1:800	Thermo Fisher Scientific, Waltham, MA	AB_2535864
Anti-Guinea pig IgG-conjugated to Alexa Fluor 488	706-545-148	Donkey	Polyclonal	1:800	Jackson ImmunoResearch Labs, West Grove, PA	AB_2340472
Anti-Guinea pig IgG-conjugated to Alexa Fluor 647	706-605-148	Donkey	Polyclonal	1:800	Jackson ImmunoResearch Labs, West Grove, PA	AB_2340476
Anti-rabbit IgG-conjugated to biotin	BA-1000	Goat	Polyclonal	1:200	Vector Laboratories, Burlingame, CA	AB_2313606

*RRID* research resource identifier

The sections for bright-field observations were incubated with 3% normal rabbit serum and 0.2% Triton X-100 in PBS for 1 h; primary antibodies were incubated in the same incubation buffer overnight, and secondary antibodies were incubated in the same incubation solution for 3 h. Then the sections were incubated in 1:100 avidin–biotin–peroxidase complex (PK-4000, Vector Laboratories, Burlington, CA, USA) in PBS for 1 h. Subsequently, the sections were reacted with 0.02% diaminobenzidine tetrahydrochloride (D5637, Merck, Darmstadt, Germany) and 0.005% H_2_O_2_ in 0.05 M Tris–HCl buffer for 20 min.

Finally, the sections were mounted on glass slides (Platinum Pro; Matsunami Glass, Osaka, Japan), air-dried, and coverslipped. Photomicrographs of the ganglion sections were taken using a digital slide scanner (BZ-X700; Keyence, Osaka, Japan) or a confocal laser scanning microscope (LSM 700; Carl Zeiss Microscopy, Jena, Germany).

### Electron microscopy

The trigeminal ganglion was removed, cut into 50 µm sections with a micro slicer (DSK-2000; Dosaka EM, Kyoto, Japan), postfixed in the same fixative solution, and kept overnight at 4 °C. The sections were treated with 1% osmium tetroxide solution for 30 min, dehydrated, and embedded in epoxy resin (a mixture of Luveak-812, DDSA, MNA, and DMP-30). Then 1 µm semi-thin sections were prepared using an ultramicrotome (EM UC7; Leica Microsystems, Wetzlar, Germany) and stained with 0.5% toluidine blue solution to define the observation area. Next, 70 nm ultra-thin sections were prepared, placed on Formvar-coated grids, and stained with 1% uranyl acetate solution for 30 min and Reynolds’ lead citrate solution for 5 min. Photomicrographs of the ultra-thin sections were taken using a transmission electron microscope (H-7650; Hitachi High-Tech, Tokyo, Japan).

### Data analysis

The region of interest was defined as the area with dense neurons in the form of islands, excluding the nerve fibers in the maxillary nerve region of the trigeminal ganglion. Multiple regions of interest were extracted from three or more trigeminal ganglion sections and averaged to obtain *n* = 1. Measurements of ganglionic sections were analyzed using Fiji/ImageJ software (NIH, Bethesda, MD, USA). Three-dimensional reconstructed images were drawn using Neurolucida software (MBF Bioscience, Williston, VT, USA) from photomicrographs taken under a confocal laser scanning microscope. The statistical analysis employed Graphpad Prism 7 software (GraphPad Software, La Jolla, CA, USA). Data are expressed as mean ± standard deviation, and differences were detected using one-way or two-way factorial analysis of variance (ANOVA) with the Tu–Kramer post hoc test or the unpaired Student’s *t* test with Welch’s correction. A *P* value < 0.05 was considered significantly different.

## Results

### Proliferation of ganglionic macrophages after nerve injury

To evaluate neuronal damage and cellular activity of macrophages in sensory ganglia after nerve injury over time, the maxillary nerve region of the trigeminal ganglion was immunohistochemically stained after ligating the infraorbital nerve.

**Fig. 1:**
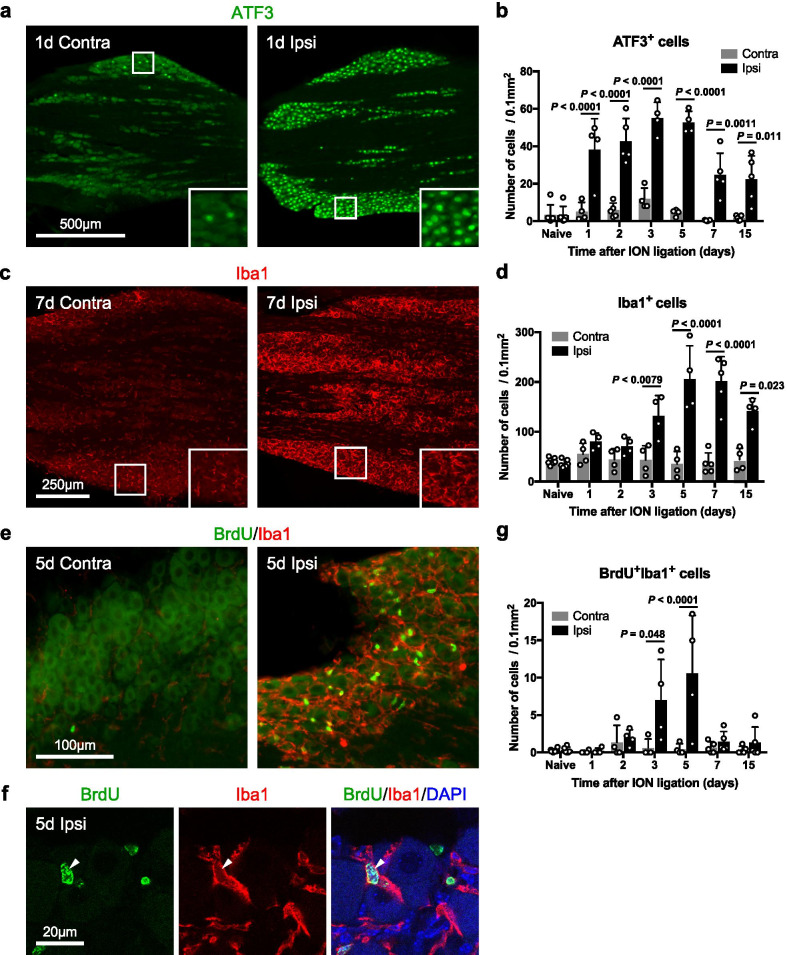
Ganglionic macrophages proliferate after nerve injury. ATF3-positive cells (green) in the contralateral (contra) and ipsilateral (ipsi) sides of the maxillary nerve region of the trigeminal ganglion on day 1 after infraorbital nerve ligation (**a**) and the number of ATF3-positive cells (*n* = 4–6/timepoints) (**b**). Iba1-positive cells (red) on day 7 after nerve ligation (**c**) and the number of Iba1-positive cells (*n* = 4–6/timepoints) (**d**). BrdU (green)- and Iba1 (red)-positive cells (**e**), multiple staining showing co-localization (arrowhead) of BrdU signals (green) with nucleus (blue) of Iba1-positive cells (red) (**f**) on day 5 after a nerve ligation, and the number of BrdU- and Iba1-positive cells (*n* = 4–6/timepoints) (**g**). See list of abbreviations. Scale bars are indicated. Data are represented as mean (S.D.), and differences were detected using two-way ANOVA with Tukey–Kramer test (**b**, **d**, **g**)

Activating transcription factor 3 (ATF3) is a marker of damaged neurons [26] and is also involved in axonal regeneration, as axonal outgrowth is reduced in ATF3-deficient mice [27, 28]. Therefore, ATF3 was used to identify the location to observe in the maxillary nerve region of the trigeminal ganglion and to confirm the expression of ATF3 in the time series. After the infraorbital nerve was ligated, ATF3-positive cells were detected over a wide range of the maxillary nerve region on the ipsilateral side (Fig. 1a, Additional file 1: Figure S1a–c). By contrast, the mandibular nerve region in the ipsilateral trigeminal ganglion had almost no ATF3-positive cells (Additional file 1: Figure S1a, d). Therefore, the entire maxillary nerve region was used as our observation point. The numbers of ATF3-positive cells increased significantly on the ipsilateral side of the maxillary nerve region compared to the contralateral side from day 1 to day 15 after infraorbital nerve ligation (Fig. 1a, b).

The macrophage/microglia marker Iba1 [29, 30] was used to examine changes in the numbers of macrophages in a time series. After the infraorbital nerve was ligated, the numbers of Iba1-positive cells increased significantly on the ipsilateral side of the maxillary nerve region compared to the contralateral side from day 3 to day 15 (Fig. 1c, d).

The cell-proliferation marker BrdU was administered as a single dose to nerve-ligated mice 24 h before perfusion. After infraorbital nerve ligation, numbers of BrdU- and Iba1-positive cells increased significantly on the ipsilateral side of the maxillary nerve region compared to the contralateral side from day 3 to day 5 (Fig. 1e–g). These results indicate that primary sensory neurons had increased ATF3 activity on day 1 after nerve injury, and then ganglionic macrophages underwent cell proliferation from days 3 to 5 and cell numbers increased from days 3 to 15.

### Migration of ganglionic macrophages after nerve injury

To elucidate whether proliferated macrophages in sensory ganglia after nerve injury are tissue-resident or BMD, we transected the infraorbital nerve of head-protected GFP-BMT mice and looked for GFP-positive BMD cells in the maxillary nerve region of the trigeminal ganglion.

**Fig. 2:**
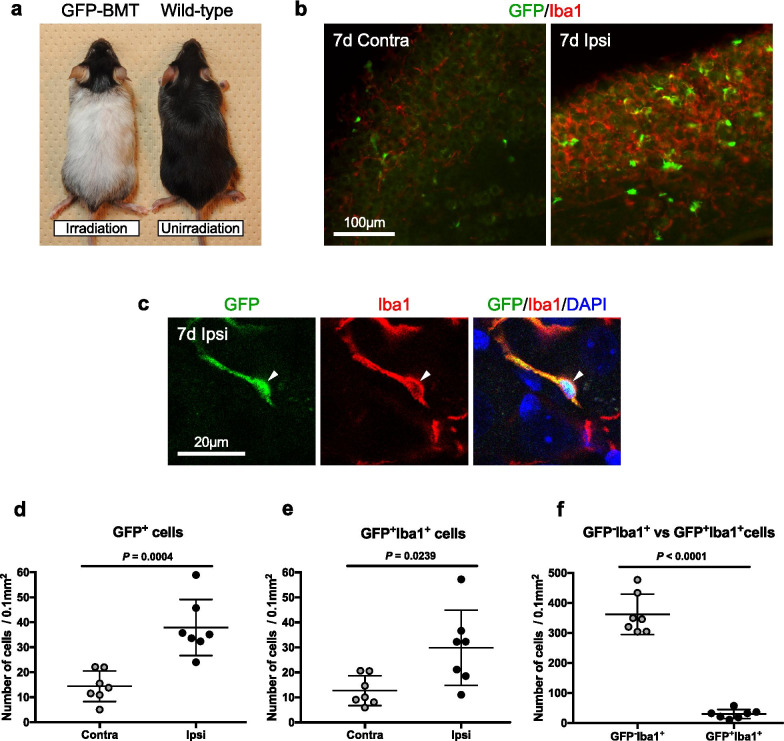
BMD macrophages increase in sensory ganglia after nerve injury. GFP-BMT mice after being irradiated with a lead cap and unirradiated wild-type mice. Irradiated GFP-BMT mice after wearing a lead cap and unirradiated wild-type mice (**a**). GFP (green)- and Iba1 (red)-positive cells (**b**) and multiple staining showing co-localization (arrowhead) of GFP signals (green) with Iba1-positive cells (red) (**c**) in the contralateral (contra) and ipsilateral (ipsi) sides of the maxillary nerve region of the trigeminal ganglion on day 7 after nerve transection. The number of GFP-positive cells (*n* = 7/group) (**d**), GFP- and Iba1-positive cells (*n* = 7/group) (**e**), and GFP-negative and Iba1-positive cells or GFP- and Iba1-positive cells (*n* = 7/group) (**f**) on day 7 after nerve transection. See list of abbreviations. Scale bars are indicated. Data are represented as mean (S.D.), and differences were detected using Student’s *t* test (**d**) and Student’s *t* test with Welch’s correction (**e**, **f**)

After radiation exposure, macrophages in the dorsal horn of the spinal cord migrate from blood vessels due to disruption of the blood–brain barrier [12]. Therefore, in this study, the head, including the trigeminal ganglion, was protected with a lead cap to prevent direct radiation exposure. The body of head-protected GFP-BMT mice turned white and the head turned black (Fig. 2a).

Macrophages in sensory ganglia are thought to originate from bone marrow [11]. To confirm this, we created a nerve-injury model in 7-month-old mice, 5 months after transplanting BMD cells. As the macrophages barely proliferated and cell numbers peaked and remained constant on day 7 after nerve injury (Fig. 1c–g), we chose day 7 as our observation point. Because a previous study using GFP-BMT mice prepared a nerve crush lesion model [11], we employed infraorbital nerve transection to damage the entire infraorbital nerve rather than a part of it. After transecting the infraorbital nerve, the numbers of GFP-positive cells increased significantly on the ipsilateral side of the maxillary nerve region compared to the contralateral side by day 7 (Fig. 2b, d). Similarly, the numbers of GFP- and Iba1-positive cells increased significantly on the ipsilateral side by day 7 after nerve transection (Fig. 2b, c, e). Furthermore, GFP-negative and Iba1-positive cells made up the majority of cells (Fig. 2b, f). These results imply that although the number of BMD macrophages increased after nerve injury, most of the ganglionic macrophages were tissue-resident.

### Morphological changes in ganglionic macrophages after nerve injury

To investigate changes in the form of macrophages in sensory ganglia after nerve injury, we attempted to three-dimensionally reconstruct macrophages in the maxillary nerve region of the trigeminal ganglion after ligating the infraorbital nerve.

**Fig. 3:**
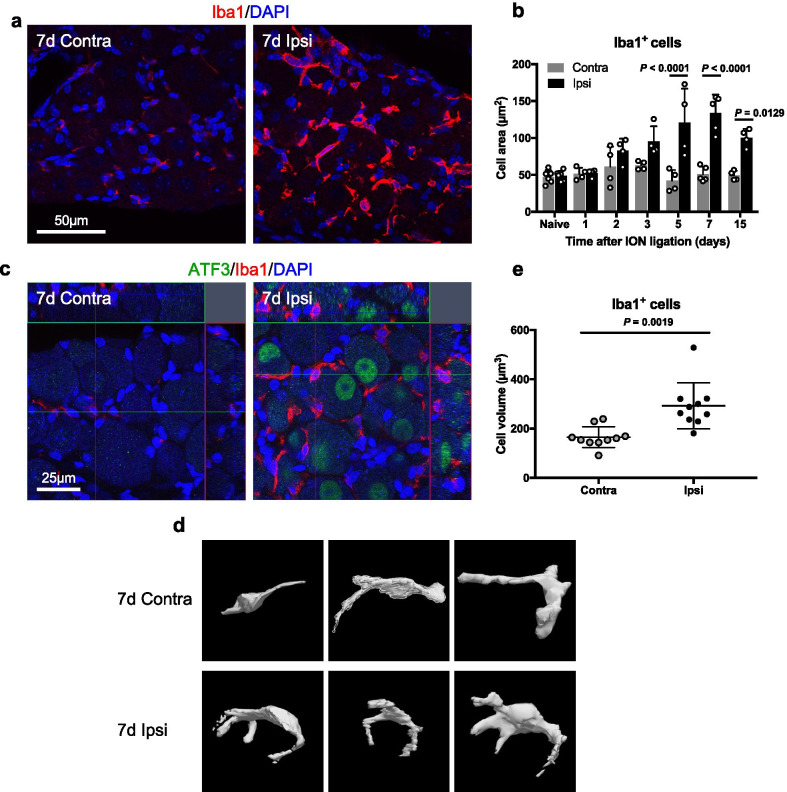
Volume of ganglionic macrophages enlarges after nerve injury. Iba1-positive cells (red) in the contralateral (contra) and ipsilateral (ipsi) sides of the maxillary nerve region of the trigeminal ganglion on day 7 after infraorbital nerve ligation (**a**) and the cell areas of Iba1-positive cells (*n* = 4–6/timepoints) (**b**). Z-stack images showing Iba1-positive cells (red) around ATF3-positive cells (green) (**c**), three-dimensional images showing Iba1-positive cells (**d**), and the cell volume of Iba1-positive cells (*n* = 10 cells/group from 3 mice) (**e**) on day 7 after nerve ligation. See list of abbreviations. Scale bars are indicated. Data are represented as mean (S.D.), and differences were detected using two-way ANOVA with Tukey–Kramer test (**b**) and Student’s *t* test with Welch’s correction (**e**)

Before reconstruction, we examined the increase in the cell area of ganglionic macrophages over time after ligating the infraorbital nerve. That of Iba1-positive cells increased significantly on the ipsilateral side of the maxillary nerve region compared to the contralateral side from day 5 to day 15 after infraorbital nerve ligation (Fig. 3a, b).

Subsequently, the volume of macrophages around damaged neurons was measured on day 7, when the cell area of macrophages was at its maximum after ligation. After ligation, the cell volume of Iba1-positive cells surrounding ATF3-positive cells on the ipsilateral side increased significantly compared to Iba1-positive cells surrounding ATF3-negative cells on the contralateral side (Fig. 3c–e). In addition, Iba1-positive cells appeared to extend multiple processes that surrounded ATF3-positive cells (Fig. 3d).

**Fig. 4:**
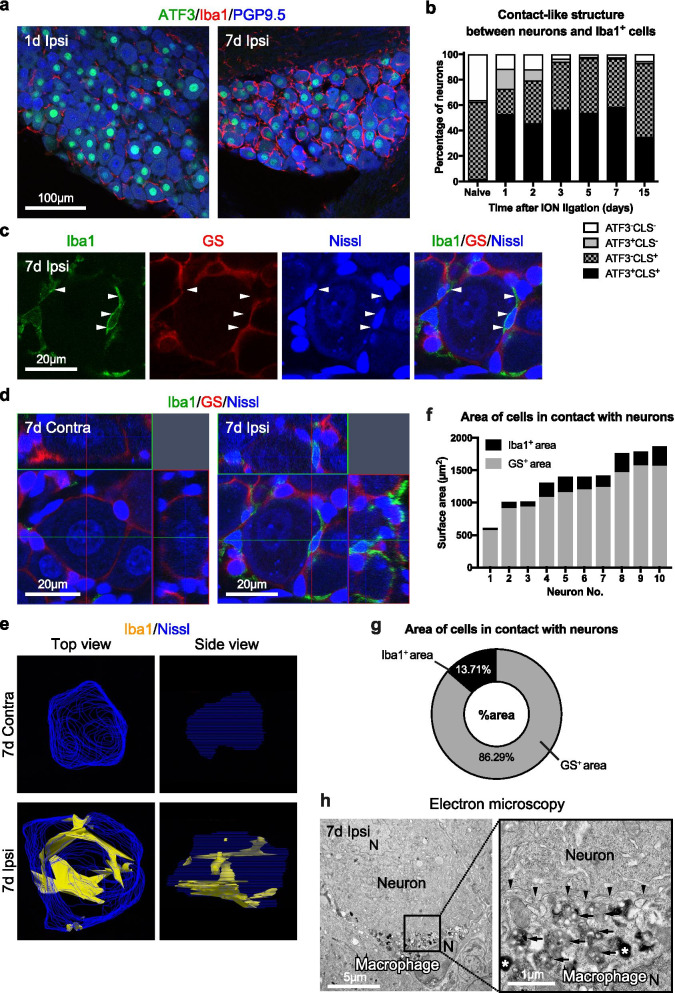
Contact areas of ganglionic macrophages to primary sensory neurons are expanded after nerve injury. ATF3 (green)-, Iba1 (red)-, and PGP9.5 (blue)-positive cells in ipsilateral (ipsi) sides of the maxillary nerve region of the trigeminal ganglion on days 1 and 7 after infraorbital nerve ligation (**a**) and a percentage of contact-like structures (CLS) between ATF3-positive or negative neurons and Iba1-positive cells (*n* = 4 or 5/group) (**b**). Multiple staining (**c**) and Z-stack images (**d**) showing Iba1-positive cells (green), Nissl-positive neurons (blue), and glutamine synthetase (GS)-positive satellite glial cells (red) in the contralateral (contra) and ipsilateral sides on day 7 after nerve ligation. White arrowhead indicates the contact sites between Iba1-positive cells and neurons. Three-dimensional images showing contact area of Iba1-positive cells and Nissl-positive neurons (**e**). Blue indicates the surface of Nissl-positive neurons, and yellow indicates the surface of Nissl-positive neurons with Iba1-positive cells in contact. Surface area of Iba1-positive or GS-positive satellite glial cells in contact with individual Nissl-positive neurons (**f**) and a percentage of these areas averaged together (averaging 10 cells/ipsi from 3 mice) (**g**) on day 7 after nerve ligation. Electron micrograph showing macrophages with electron-dense lipid bodies (asterisk) and lysosomes (arrow) in contact (black arrowhead) with neurons (**h**). “N” indicates the nucleus. See list of abbreviations. Scale bars are indicated

Before the three-dimensional reconstruction of contact relationships among macrophages, neurons, and satellite glial cells after infraorbital nerve ligation using high-magnification images, we defined “a contact-like structure” as a structure in which macrophages appear to be in contact with neurons using low-magnification images, and studied this structure over time. We used ATF3, Iba1, and product gene protein 9.5 (PGP9.5; a neuron marker) as indicators and prepared a simplified classification as follows: ATF3-positive neurons form a contact-like structure with Iba1-positive cells (ATF3^+^CLS^+^ neurons), ATF3-negative neurons form a contact-like structure with Iba1-positive cells (ATF3^−^CLS^+^ neurons), ATF3-positive neurons do not form a contact-like structure with Iba1-positive cells (ATF3^+^CLS^−^ neuron), and ATF3-negative neurons do not form a contact-like structure with Iba1-positive cells (ATF3^−^CLS^−^ neuron). Most of the neurons showed contact-like structures with Iba1-positive cells from day 5 to day 7 after infraorbital nerve ligation (Fig. 4a, b).

In naïve mice, neurons in sensory ganglia are covered with satellite glial cells [20]. To ascertain whether ganglionic macrophages contact the neurons and to determine the area of contact after nerve injury, we created three-dimensional reconstructed images from the immunohistochemistry results, and measured the contact areas between neurons and macrophages or satellite glial cells. We defined Iba1-positive cells, which are located between neurons and glutamine synthetase (a marker of satellite glial cell)-positive cells, as macrophages contacting neurons (Fig. 4c). Reconstruction was performed on day 7, when the contact-like structures between macrophages and neurons were observed (Fig. 4a, b) and the area of macrophages was at its maximum (Fig. 3a, b). Neurons were covered with glutamine synthetase-positive satellite glial cells, and Iba1-positive cells did not contact neurons on the contralateral side on day 7 after infraorbital nerve ligation (Fig. 4d, e), whereas Iba1-positive cells directly contacted the neurons on the ipsilateral side (Fig. 4c–e). The percentage of surface area, where Iba1-positive cells were in contact with neurons was about 13.71% (Fig. 4f, g). Furthermore, macrophages have electron-dense lipid bodies and lysosomes in their cytoplasm [31, 32]. The microstructure confirmed that macrophages with abundant lipid bodies and lysosomes contacted the neurons on day 7 after infraorbital nerve ligation (Fig. 4h). These results indicate that ganglionic macrophages increase in volume, and directly contact damaged neurons via multiple processes after nerve injury.

### Classification of ganglionic macrophages after nerve injury

**Fig. 5:**
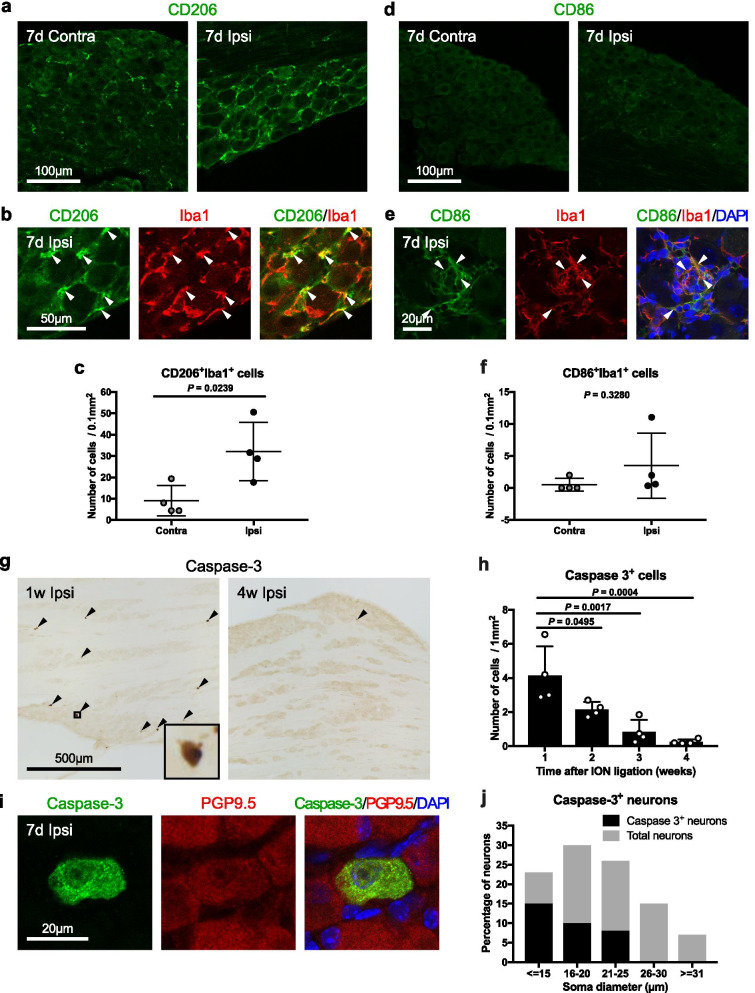
Ganglionic macrophages in sensory ganglia after nerve injury are categorized to “tissue repair”. CD206-positive cells (green) (**a**), multiple staining showing co-localization (arrowhead) of CD206 signals (green) with Iba1-positive cells (red) (**b**), and the number of CD206- and Iba1-positive cells (*n* = 4/group) (**c**) in contralateral (contra) and ipsilateral (ipsi) sides of the maxillary nerve region of the trigeminal ganglion on day 7 after infraorbital nerve ligation. CD86-positive cells (green) (**d**), multiple staining showing co-localization (arrowhead) of CD86 signals (green) with Iba1-positive cells (red) (**e**), and the number of CD86- and Iba1-positive cells (*n* = 4/group) (**f**) on day 7 after infraorbital nerve ligation. Caspase-3-positive cells (brown, arrowhead) on weeks 1 and 4 after nerve ligation (**g**) and the number of caspase-3-positive cells (*n* = 4/timepoint) (**h**). Caspase-3 (green)- and PGP9.5 (red)-positive cells (**i**) and the percentage of caspase-3-positive neurons (*n* = 129 cells/ipsi from 4 mice) (**j**) on day 7 after nerve ligation. See list of abbreviations. Scale bars are indicated. Data are represented as mean (S.D.), and differences were detected using Student’s *t* test (**c**), Student’s *t* test with Welch’s correction (**f**), and one-way ANOVA with Tukey–Kramer test (**h**)

Macrophages are broadly classified into the M1 or M2 phenotypes [21]. To determine whether the macrophages that contacted neurons were of the M1 or M2 phenotype in sensory ganglia on day 7 after nerve injury, we investigated CD86, an M1 phenotype marker, and CD206, an M2 phenotype marker, in the maxillary nerve region of the trigeminal ganglion after ligating the infraorbital nerve. The numbers of CD206- and Iba1-positive cells increased significantly on the ipsilateral side compared to the contralateral side (Fig. 5a–c). By contrast, the numbers of CD86- and Iba1-positive cells did not increase significantly (Fig. 5d, f), but a zone rich in CD86- and Iba1-positive cells was observed in some areas (Fig. 5e).

Then we examined whether contact between ganglionic macrophages and neurons was associated with neuronal cell death after nerve injury. Contact between macrophages and neurons was seen on day 7 after nerve injury (Fig. 4c–g), and a previous study that used Western blotting showed that caspase-3, a marker of cell death, is elevated from week 1 to week 4 after nerve injury [33]. Therefore, to determine the number of dying neurons after nerve injury, we performed a histological study using cleaved caspase-3. Caspase-3-positive cells were detected on the ipsilateral side at 1 week after infraorbital nerve ligation (Fig. 5g) and then decreased in number at from 2 to 4 weeks after infraorbital nerve ligation (Fig. 5h).

Small sensory ganglia neurons are more likely to die after nerve injury [34, 35]. Therefore, we measured neuron size to determine whether caspase-3-positive cells were more common in small or large neuron types. The soma size of caspase-3- and PGP9.5-positive neurons was < 25 µm at 1 week after infraorbital nerve ligation (Fig. 5i, j). These results show that ganglionic macrophages exhibited the M2 phenotype on day 7 after nerve injury when macrophages were in contact with neurons, and that cell death occurred in a small number of damaged small neurons but not in most damaged neurons.

## Discussion

Macrophages in sensory ganglia are essential for controlling inflammation, nerve tissue repair and neuropathic pain after peripheral nerve injury. We showed that ganglionic macrophages, which proliferate rapidly after nerve injury, are tissue-resident, enter between satellite glial cells and neurons, are in direct contact with neurons, and exhibit the M2 phenotype with almost no neuronal cell death, indicating that this contact may be related to tissue repair. Until now, whether ganglionic macrophages that increase after peripheral nerve injury are tissue-resident or BMD has been studied only indirectly, such as with macrophage-depleting agents, and no direct evidence has been reported. In addition, while morphological changes in microglia after peripheral nerve injury are an indicator of activation, ganglionic macrophages have very limited morphological information, including a three-dimensional structure. Our study was designed to compensate for this limitation.

### Macrophages proliferate in sensory ganglia after nerve injury

We identified a time series of phenomena associated with inflammation in sensory ganglia, such as an increase in the numbers of damaged neurons from day 1 after peripheral nerve injury followed by division of macrophages from days 3 to 5 and an increase in the numbers of macrophages on days 3–15. Previous histological studies on ATF3 in sensory ganglia after nerve injury have reported a rapid increase in ATF3-positive cells from day 1 [26, 36]. This trend was also observed in an investigation of ATF3-positive cell numbers in the facial nucleus after nerve injury [28]. It was individually reported that macrophages in sensory or autonomic ganglia proliferate 2–4 days after nerve injury using BrdU or Ki67 [5, 37, 38]. In a preliminary study using Ki67 by Krishnan et al. [5], although there was no significant difference, the increase began on day 2, peaked on day 3, and showed a decreasing trend after day 7. Lu and Richardson [39] reported that, after nerve injury, ganglionic macrophages increase within 4 days and persist for more than 1 month. Kwon et al. [6] reported an increase in the numbers of ganglionic macrophages from 3 to 28 days after nerve injury. Our results support these findings. In brief, ganglionic macrophages proliferate and increase rapidly after neurons are damaged due to nerve injury, and this state seems to be maintained.

### BMD macrophages increase in sensory ganglia after nerve injury, but most macrophages are tissue-resident

We found that BMD macrophages increased in sensory ganglia after nerve injury, but the majority of ganglionic macrophages were tissue-resident. In a previous study of physiological turnover using bone-marrow-transplanted mice in sensory ganglia, 80% of cells were replaced by BMD macrophages within 3 months [11]. However, under the influence of radiation BMD macrophages infiltrate the central nervous system [12], so we protected the head of mice with a lead cap during irradiation and observed the trigeminal ganglion located in the lower part of the brain. In this way, we determined that most of the ganglionic macrophages were tissue-resident in mice at 5 months after cell transplantation, implying that the turnover of BMD cells in sensory ganglia may be due to radiation damage. This result supports reports that the use of the macrophage-depleting agent clodronate does not change the numbers of macrophages in sensory ganglia but reduces the numbers of monocytes in the blood [3, 5]. Considering that tissue-resident macrophages in the central nervous system are microglia [8], macrophages in the sensory ganglia of the peripheral nervous system may be tissue-resident. Nevertheless, an increase in BMD macrophages has also been observed after nerve injury. Cobos et al*.* [40] reported a decrease in mRNA for the monocyte/macrophage markers CD68, CD11b, and CD 163 in sensory ganglia after nerve injury by clodronate treatment. The decrease in these markers implies that some macrophages in sensory ganglia are BMD macrophages. Furthermore, a study using parabiosis showed that the majority of macrophages are tissue-resident, although a small influx of hematogenous leukocytes is observed in sensory ganglia after nerve injury [17]. In summary, our results indicate that a large number of tissue-resident macrophages and a small number of BMD macrophages are mixed in sensory ganglia after nerve injury. BMD macrophages express chemokine (CC motif) receptor 2, and these deficient mice suppress the increase in ganglionic macrophages after nerve injury [41], suggesting that a small number of BMD macrophages may trigger peripheral nerve regeneration or neuropathic pain after nerve injury.

### Morphological changes in macrophages from sensory ganglia after nerve injury

We found that the area of macrophages increased from day 5 to day 15 after nerve injury. In addition, we also extracted and three-dimensionally reconstructed macrophages after nerve injury on day 7 and found that the cytoplasm of macrophages around damaged nerve cells increased. Macrophages in sensory ganglia reportedly increase in volume in hemiplegic migraine [16] and nerve injury models [17], and this study showed similar findings. Moreover, the shape of the macrophages is amoeboid in the migraine model [16], while they show a stellate shape in the nerve injury model [17]. Our results support the latter, with macrophages extending their processes to surround the neurons. We analyzed the ganglionic macrophages adjacent to ATF3-positive neurons, and these morphological differences may have been due to differences in the models used and the sites measured.

Most of the neurons showed contact-like structures with macrophages from days 5 to 7 after nerve injury. These structures were observed in damaged and undamaged neurons. Furthermore, we extracted and three-dimensionally reconstructed the cells on day 7 after nerve injury and found that the average contact area of macrophages to the neuronal surface was 13.71% (Fig. 4g). Kwon et al. [6] reported that approximately 75% of ganglionic macrophages are in contact with neurons after nerve injury. In addition, we showed that the contact-like structures of macrophages extended to undamaged neurons. Damaged and undamaged neurons have electrical coupling via gap junctions between satellite cells [20]. In addition, after nerve injury, chemokine (CC motif) ligand 2/monocyte chemoattractant protein 1 is elevated in primary sensory neurons, and macrophage accumulation does not occur in these deficient mice [7]. These findings suggest that hyperexcitable damaged neurons and adjacent undamaged neurons release chemokine and promote macrophage contact. Neurons in sensory ganglia that have not been injured are covered with satellite glial cells [20], and in our study, neurons were surrounded by satellite glial cells in the three-dimensional reconstruction of the sham side. By contrast, neurons on the injured side were not completely covered by satellite glial cells; macrophages partially entered between neurons and satellite glial cells, and neurons and macrophages came in contact with each other. In the central nervous system, contact between microglia and the cell bodies of neurons is protective, inhibiting the over-activity that occurs during neuronal injury [42, 43]. It is unclear whether these same functions are present in ganglionic macrophage–neuron contacts, and further investigation is needed.

### Macrophages presented the M2 phenotype in sensory ganglia after nerve injury

Ganglionic macrophages showed M2 phenotype activity in sensory ganglia and a small number of small neurons died after the nerve injury. Upregulation of the M2 markers CD206 and arginase-1 after nerve injury has been reported previously [7, 22–24]. The expression of CD206 in our study was similar to these previous results. However, although M1 marker CD86-positive macrophages were not conspicuous throughout sensory ganglia, CD86-positive macrophages were partially accumulated. Niemi et al. [22] reported an increase in CD86 mRNA along with CD206, and their data represent the small number of CD86-positive macrophages observed in this study. The significance of the rare accumulation of CD86-positive macrophages is unclear and awaits further investigation.

If we assume that most ganglionic macrophages after nerve injury are neuroprotective, neuronal cell death may be unlikely to occur. In previous studies, a significant decrease in the numbers of cells was observed at 7 days after nerve injury [44], whereas neuronal cell death was not observed [45, 46] or was observed only in a few neurons by TUNEL assay [47, 48]. In this study, we used caspase-3 and detected a small number of caspase-3-positive neurons; these cells decreased from weeks 1 to 4. This same decrease was also observed in western blotting of sensory ganglia [33]. The numbers of caspase-3-positive neurons were considerably lower than the numbers of ATF3-positive neurons after nerve injury. It is conceivable that most damaged neurons do not die after distal peripheral nerve injury, such as that in this study. On the other hand, some caspase-3-positive cells were observed until at least 3 weeks, implying that cells died gradually. In addition, caspase-3 tended to be detected in small neurons. Previous studies have found that small cells are more likely to die after nerve injury [34, 35] and unmyelinated fibers are more likely to disappear than are myelinated fibers [34, 49]. Furthermore, Vega-Avelaira et al. [18] reported that many ganglionic macrophages around C-neurons (< 25 µm) do not have ring-like structures, whereas A-neurons (> 25 µm) do. These findings suggest that the contact of M2 phenotype macrophages contributes to protecting neurons from neuronal cell death rather than phagocytosing neurons after nerve injury.

## Conclusion

We found that, in sensory ganglia, macrophages proliferate for several days after neurons are damaged, most macrophages in ganglia are tissue-resident, macrophages become larger and directly contact damaged neurons, most macrophages are of the M2 phenotype, and neuronal cell death rarely occurs. The significance of the contact of macrophages with neurons is unknown and awaits further study.

## Supplementary Information

**Additional file 1: Figure S1.** ATF3-positive cells (green) in the ipsilateral (ipsi) side of the trigeminal ganglion on day 1 after infraorbital nerve ligation are shown. The maxillary nerve region (the left side of magenta dashed line) and the mandibular nerve region (the right side of magenta dashed line) (a) and their magnified views (b–d). See list of abbreviations. Scale bars are indicated.

## Data Availability

The data sets used and/or analysed during the current study are available from the corresponding author on reasonable request.

## Abbreviations

ATF3: Activating transcription factor 3
BrdU: Bromodeoxyuridine
BMD: Bone marrow-derived
CD: Cluster of differentiation
CLS: Contact-like structure
Contra: Contralateral
DAPI: 4ʹ,6-Diamidino-2-phenylindole
GFP: Green fluorescent protein
GFP-BMT: Green fluorescent protein-positive bone marrow cells-transplanted
GFP-Tg: Green fluorescent protein-transgenic
Iba1: Ionized calcium-binding adaptor molecule 1
Ipsi: Ipsilateral
PBS: Phosphate buffered saline
N: Nucleus
PGP9.5: Product gene protein 9.5
