# Supplementary figures and images for: Tissue-resident M2 macrophages directly contact primary sensory neurons in the sensory ganglia after nerve injury
Source: J Neuroinflammation. 2021 Oct 13;18:227. doi: 10.1186/s12974-021-02283-z (PMC8513227; doi:10.1186/s12974-021-02283-z)

**a**

ATF3

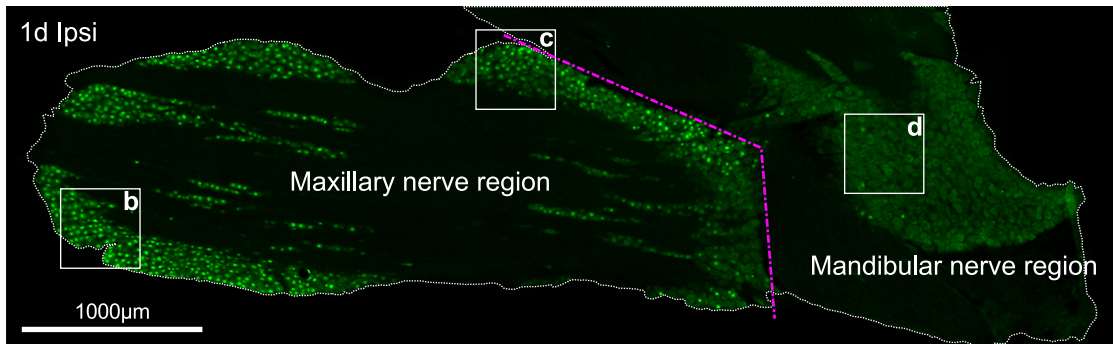**b**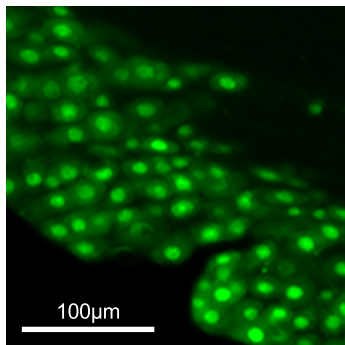**c**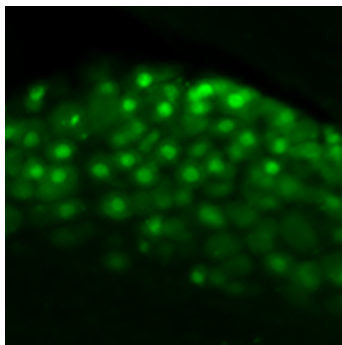**d**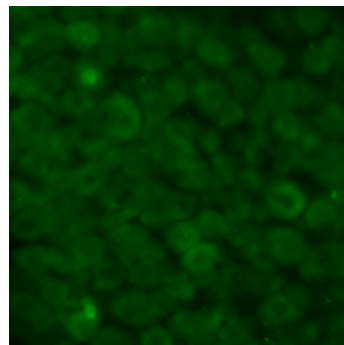

Supplement: Supplementary file 1 — Additional file 1: Figure S1. ATF3-positive cells (green) in the ipsilateral (ipsi) side of the trigeminal ganglion on day 1 after infraorbital nerve ligation are shown. The maxillary nerve region (the left side of magenta dashed line) and the mandibular nerve region (the right side of magenta dashed line) (a) and their magnified views (b–d). See list of abbreviations. Scale bars are indicated. [file 12974_2021_2283_MOESM1_ESM.pdf]
